# Polymorphic Variants of SCN5A Gene (rs41312433 and rs1805124) Associated with Coronary Artery Affliction in Patients with Severe Arrhythmias

**DOI:** 10.3390/genes15020200

**Published:** 2024-02-02

**Authors:** Anna Vašků, Tomáš Novotný, Jindřich Špinar

**Affiliations:** 1 Department of Pathophysiology, Faculty of Medicine, Masaryk University, 62500 Brno, Czech Republic; 2 Department of Internal Medicine and Cardiology, University Hospital Brno, Masaryk University, 62500 Brno, Czech Republic; novotny.tomas3@fnbrno.cz; 3 First Department of Internal Medicine—Cardioangiology, St. Anne’s University Hospital, Faculty of Medicine, Masaryk University, 60200 Brno, Czech Republic; jspinar@med.muni.cz

**Keywords:** coronary artery disease, arrhythmia, SCN5A

## Abstract

Several mutations in this gene for the α subunit of the cardiac sodium channel have been identified in a heterogeneous subset of cardiac rhythm syndromes, including Brugada syndrome, progressive cardiac conduction defect, sick sinus node syndrome, atrial fibrillation and dilated cardiomyopathy. The aim of our study was to associate some SCN5A polymorphic variants directly with confirmed coronary stenoses in patients with non-LQTS ventricular fibrillation/flutter treated by an implantable cardioverter defibrillator. Materials and Methods: A group of 32 unrelated individuals, aged 63 ± 12 years, was included in the study. All the patients were examined, diagnosed and treated with an implantable cardioverter defibrillator at the Department of Internal Cardiology Medicine, Faculty Hospital Brno. The control group included 87 persons of similar age without afflicted coronary circulation, which was confirmed coronagraphically. Genomic DNA was extracted from samples of peripheral blood according to the standard protocol. Two SCN5A polymorphisms—IVS9-3C/A (rs41312433) and A1673G (rs1805124, H558R)—were examined in association with coronary artery stenosis in the patients. Results: In the case–control study, no significant differences in genotype distribution/allelic frequencies were observed for IVS9-3c>a and A1673G gene polymorphisms between patients with severe arrhythmias and healthy persons. The distribution of SCN5A double genotypes was not significantly different among different types of arrhythmias according to their ejection fraction in arrhythmic patients (*p* = 0.396). The ventricular arrhythmias with an ejection fraction below 40% were found to be 10.67 times more frequent in patients with multiple coronary stenosis with clinically valid sensitivity, specificity and power tests. In the genotype–phenotype study, we observed a significant association of both SCN5A polymorphisms with the stenosis of coronary vessels in the patients with severe arrhythmia. The double genotype of polymorphisms IVS9-3C/A together with A1673G (CCAA) as well as their simple genotypes were associated with significant multiple stenosis of coronary arteries (MVS) with high sensitivity and specificity (*p* = 0.05; OR = 5 (95% CI 0.99–23.34); sensitivity 0.70; specificity 0.682; power test 0.359) Moreover, when a concrete stenotic coronary artery was associated with SCN5A genotypes, the CCAA double genotype was observed to be five times more frequent in patients with significant stenosis in the right coronary artery (RCA) compared to those without affliction of this coronary artery (*p* = 0.05; OR = 5 (95% CI 0.99–23.34); sensitivity 0.682; specificity 0.700; power test 0.359). The CCAA genotype was also more frequent in patients without RCA affliction with MVS (*p* = 0.008); in patients with ACD affliction but without MVS (*p* = 0.008); and in patients with both ACD affliction and MVS compared to those without ACD affliction and MVS (*p* = 0.005). Conclusions: Our study presents a highly sensitive and specific association of two polymorphisms in SCN5A with significant coronary artery stenoses in patients with potentially fatal ventricular arrhythmias. At the same time, these polymorphisms were not associated with arrhythmias themselves. Thus, SCN5A gene polymorphic variants may form a part of germ cell gene predisposition to ischemia.

## 1. Introduction

Sudden death is related to the development of malignant arrhythmias as ventricular fibrillation or ventricular tachycardia. Coronary artery disease (CAD) is supposed to be the most frequent factor responsible for their occurrence [1]. But other myocardial diseases can lead to the risk state for sudden death. The potential genetic predisposition of these states will be complex; polymorphic germ cell alleles and epigenetic modification in interaction with the environment can form a genetic predisposition for these secondary arrhythmias. On the other side, rare diseases without organic damage of the heart but with primary electrical abnormality can also lead to a higher risk of severe arrhythmias [1]. These diseases copy Mendelian inheritance and are associated with rare alleles in many genes coding for ion channels. The genetic base of these channelopathies is strong; mainly loss-of-function alleles with decreased sodium, potassium and calcium channels are responsible for the higher risk of severe arrhythmias in these diseases [2].

Since the identification of the first SCN5A mutation associated with long QT syndrome in 1995, several mutations in this gene for the α subunit of the cardiac sodium channel have been identified in a heterogeneous subset of cardiac rhythm syndromes. They include Brugada syndrome, progressive cardiac conduction defect, sick sinus node syndrome, atrial fibrillation sudden infant death syndrome, long QT syndromes (LQT1-10), Jervelle Lange-Nielsen syndromes and dilated cardiomyopathy [3,4,5,6,7,8,9,10]. *SCN5A* encodes the cardiac voltage-gated sodium channel (Na_v_1.5). Na_v_1.5 supports the ventricular action potential (AP) and is also relevant for the conduction of the electrical signal in the heart. The dysfunction of sodium flow can result from mutations in *SCN5A*, epigenetic modification, altered post-transcriptional and post-translational modifications and disruption of the interaction with auxiliary proteins [11].

Ventricular tachycardia and fibrillation (VT/VF) complicating Brugada syndrome, a genetic disorder linked to SCN5A mutations, and VF complicating acute myocardial infarction (AMI) have both been linked to phase 2 reentry. Because arrhythmogenesis characteristics seems to be similar, the contribution of SCN5A variants to VT/VF complicating AMI was studied [12]. The H558R polymorphism was found to be responsible for the possible further accentuation of a loss-of-function mutation effect in the SCN5A gene during ischemic conditions in the heart [13].

From the pathophysiological point of view, ion channels, especially K_v_1.5 channels, Na_v_ channels and K_ATP_ channels, are generally considered to be the main effectors in the regulation of coronary blood flow. They have very important roles in the adaptation of coronary blood flow to cardiomyocytes’ metabolism by changes in vascular tone. They can modulate the state of smooth muscle cells as well as of endothelial function. Recently, considering ischemic heart pathophysiology, an important role of coronary microvascular dysfunction is supported [13].

Ischemic heart disease is defined as an imbalance between the myocardial energy state and coronary blood flow. This is seen by the presence of atherosclerosis, coronary microvascular dysfunction, inflammation and vasospasm [14]. It seems that the pathophysiology of an ischemic heart is complex, with contributions of genetic and epigenetic factors.

The aim of our study was to associate some SCN5A polymorphic variants with coronary artery affliction in a special group of patients with non-LQTs ventricular fibrillation/flutter (VF) treated using an implantable cardioverter defibrillator which prevents the development of sudden cardiac death.

## 2. Methods

### 2.1. Subjects

A group of 32 unrelated individuals, 29 men and 3 women, aged 63 ± 12 years, was included in the study. All the patients were examined, diagnosed and treated at the Department of Internal Cardiology Medicine, Faculty Hospital Brno. In 87% of these patients, ventricle fibrillation was diagnosed, and the other 13% had ventricle flutter, with an ejection fraction median of 40% and a range of 15–60% (Table 1). All of them had circulatory arrest not related to the acute phase of myocardial infarction. The patients were resuscitated, stabilized and transferred to the clinic. There, they underwent cardiological examinations. In all patients, coronarography confirmed CAD. Based on echocardiography, the left ventricle ejection fraction was calculated. These persons were treated and identified in the implantable defibrillator registry of the clinic. All the patients were diagnosed with ventricular flutter/ventricular fibrillation (VF), the QTc median was 436 ms, and the range was 375–570 ms. The patients were divided into four groups: A—patients with ventricular fibrillation with an ejection fraction (EF) below 40%; A2—patients with ventricular flutter, EF below 40%; B—patients with ventricular fibrillation with an EF above 40%; and B2—patients with ventricular flutter with an ejection fraction above 40%.

In all the patients, at least one coronary artery was affected by severe coronary stenosis above 50%. In detail, 10 patients had one coronary artery stenosis (1VS); in 22 patients, two or more coronary arteries had been afflicted (MVS). In all patients, an implantable cardioverter defibrillator was decided to be applied.

The control group included 87 persons of similar age, 76 men and 11 women. They were basically investigated at the clinic and were evaluated as suitable control persons without signs of ischemia.

This study was approved by the Committee for Ethics of Medical Experiments on Human Subjects, Faculty of Medicine, Masaryk University, Brno, and was performed in adherence to the Declaration of Helsinki Guidelines. Participants gave their written informed consent, which has been archived.

### 2.2. Genomic DNA Extraction

Genomic DNA was extracted from samples of peripheral blood according to the standard protocol using a DNA BloodSpin Kit and the classic method of ethanol progression.

### 2.3. Genotyping

Originally, 34 intronic oligonucleotide primer pairs were used to amplify the coding area of the *SCN5A*.

Sequencing of the amplicons with potential variability (SSCP method) compared with the wild-type sequences deposited in NCBI (NT 022517) was performed using the ABI PRISM 310 instrument (Applied Biosystems, Waltham, MA, USA). Polymorphisms IVS9-3C/A (rs41312433) and A1673G (rs1805124, H558R) were examined in the study [15].

### 2.4. Statistics

Distributions of genotype and allelic frequencies and their differences were calculated using χ2 tests. Consistency of genotype frequencies with the Hardy–Weinberg equilibrium was tested using a χ2 test on a contingency table of observed versus predicted genotype frequencies.

The odds ratio (OR) and 95% confidence interval were calculated to estimate the risks related to detected polymorphisms. To calculate the significance of the OR, Fisher’s exact test was used. Clinical Calculator 1 from Vassar Stats was applied for the calculation of the sensitivity and specificity of results. The program package Statistica v. 14.0 (Statsoft Inc., Tulsa, OK, USA) was used.

## 3. Results

In our group of patients, we observed, besides severe arrhythmias, a lot of cases of severe stenosis in coronary arteries as well as left ventricular hypertrophy (Table 1). All patients of the group had one or more significant stenoses of the coronary arteries.

Associating arrhythmias with coronary stenosis, significant differences between the A (ventricular fibrillation, EF below 40%) + A2 (ventricular flutter, EF below 40%) and B (ventricular fibrillation, EF above 40%) + B2 (ventricular flutter, EF above 40%) groups had been found in one coronary stenosis as well as in two or three coronary artery stenoses compared to patients without them. The A + A2 arrhythmias are 10.67 times more frequent in patients with multiple coronary stenosis (these patients have an EF below 40%) with clinically valid sensitivity (0.727), specificity (0.800) and power tests (0.708, Table 2).

### 3.1. Case–Control Study

No significant differences in genotype distribution/allelic frequencies were observed in the case–control study, including IVS9-3c>a and A1673G (Table 3).

### 3.2. Genotype–Phenotype Study

We observed a significant association of both SCN5A polymorphisms with the significant stenosis of coronary vessels (Table 4). Both SCNA5A gene polymorphisms were found to be associated with two or three coronary vessel significant stenosis (MVS-1) in patients with severe arrhythmia. The CC genotype of the IVS9-3C/A polymorphism is 6.2 times more frequent in patients with MVS-1 and severe arrhythmia compared to those without significant coronary artery stenoses with high sensitivity (0.787) and specificity (0.700). Similarly, the AA genotype of the A1673G polymorphism is five times more frequent in patients with MVS-1 compared to those without multiple significant stenosis with high sensitivity (0.682) and specificity (0.700).

Even the double genotype of polymorphisms IVS9-3C/A together with A1673G (CCAA) was associated with the significant stenosis of coronary arteries with a high sensitivity (0.682) and specificity (0.700, Table 4).

Moreover, when a concrete stenotic coronary artery was associated with SCN5A genotypes, the CCAA double genotype was observed to be five times more frequent in patients with a significant stenosis in the arteria coronaria dextra compared to those without affliction of this coronary artery (*p* = 0.05; OR = 5 (95% CI 0.99–23.34); sensitivity 0.70; specificity 0. 682; power test 0.359, Table 5). The CCAA genotype was also more frequent in patients without ACD affliction with MVS (*p* = 0.008); in patients with ACD affliction but without MVS (*p* = 0.008); and in patients with both ACD affliction and MVS compared to those without ACD affliction and MVS (0.005, Table 5).

Surprisingly, the distribution of SCN5A double genotypes was not significantly different between the different types of arrhythmias (A + A2 vs. B + B2, *p* = 0.396).

## 4. Discussion

Our study presents an association of polymorphisms in SCN5A with significant coronary artery stenoses in patients with potentially fatal ventricular arrhythmias. In a meta-analysis from the data of 22 trials that included a total of 4149 patients who experienced sudden cardiac death (SCD) or had a high risk of SCD, an allelic model showed that rs11720524 encoding a subunit of the cardiac voltage-gated sodium channel in SCN5A protected against SCD. Subgroup analysis showed that rs11720524 in SCN5A protected against SCD in Europeans and Caucasians but not in Koreans. Rs1805124 (H558R evaluated in our study) in SCN5A was not significantly related to SCD in any model of inheritance. In subgroup analysis, no significant relationship between rs1805124 in SCN5A and SCD in European, Caucasian or Chinese populations was found [16]. It is questionable how our results can be compared with these ones, because our patients had both arrhythmia (in some specific proportion) and ischemia and had a high risk of sudden death, but no death occurred in the group due to the early implantation of a cardioverter defibrillator. Therefore, even a type of therapy could modify the results. The CC genotype of the rs11720524 polymorphism in the SCN5A gene was found to be more frequent in the highly heterogeneous cardiac death cohort compared to the control population. This effect was referred to be created by an association of this variant with cardiac death in chronic ischemic heart disease [17].

Arrhythmic conditions of the heart are described by ECG phenotypes. PR and QRS intervals reflect the depolarization of the heart and conduction time through the atria and to the atrioventricular node (PR) and the whole ventricle (QRS). The intervals are to some extent positively correlated. The SCN5A gene influences PR as well as QRS in a concordant direction. On the contrary, the SCN5A locus influences QRS and QT in a discordant fashion. Thus, variants that decrease QRS increase QT interval values [18].

The A1673G (H558R) polymorphism was identified in many ethnic groups [19,20]. Detailed sequencing analysis of the SCN5A gene described both rare and polymorphic variants in SCN5A genes in relation to ECG parameters describing cardiac conductivity PR and QRS [21]. In the study, three study groups have been compared: ESP—designed for the examination of genomic associations with heart, lung and bone marrow diseases. The CHARGE study sample included participants with extreme PR and QRS values. Study sample UK10K tried to associate rare variants of the SCN5A gene with PR and QRS. As a result of the meta-analysis of these three studies, rare variants in SCN5A were associated with PR in persons with European as well as African ancestry. Considering the polymorphic variants of SCN5A, rs1805124 (H558G), the G558 allele had similar frequencies in all studies (24.4%, 18.4% and 23.4%, respectively), with a highly significant association with the PR interval value (*p* = 6.2 × 10^4^, heterogeneity of *p* = 0.09). Similarly, the QRS interval value was also highly significantly associated with the R558 value in the meta-analysis of the three studies, *p* = 5.2 × 10^3^ with a higher heterogeneity of *p* = 0.21. Interestingly, the polymorpsm was associated with shorter PR as well as QRS values. Rs1805124 was found to be in linkage disequilibrium with other SCN5A polymorphisms in the SCN5A gene—rs10865879 (intergenic region of the gene) and rs11708996 (intron) [18].

The H558G polymorphism was shown to have a lot of phenotypic effects. The G allele decreased the electrophysiological effect of the T512I mutation and influenced the effect of another spontaneous germline missense mutation, M1766L, in the SCN5A gene [22]. When the sodium channel contained L1766 completed by H558, the reduction of Ina followed. When L1766 was present together with R558, the phenotype effects of L1766 were almost null [23]. Thus, arginin can be protective when some other rare alleles are inherited.

The SCN5A polymorphism H558R was shown to be a modifier that protects against VF occurrence in BrS. Considering epigenetic modification, H558R decreased the SCN5A promoter methylation and increased the expression level in cardiac tissue. An allelic expression imbalance in BrS with a heterozygous H558R may also contribute to the protective effects in heterozygous carriers [24]. Considering the potential disease modifiers of inherited SCN5A channelopathy, it is known that SCN5A gene variability can lead to many cardiac disease phenotypes which can overlap [25]. Also, non-genetic modifiers can be taken in account: age, lifestyle, medication, alcohol, smoking, recreational drug use, exercise, diet and ecotoxicology aspects. In consequence, it will also be useful to focus on potential differences between men and women in the genetic base of arrhythmic syndromes [26]. As an example, combined coronary artery disease, age, total cholesterol, left atrial diameter and the G allele of the A1673G (H558R) polymorphism were found to be associated with atrial fibrillation in Tibetan and Han populations living at high altitudes [20]. Our results for the genotype distribution are significantly different both in the control and patient groups.

The intron polymorphism IVS9-3 C/A was published by Aydin in 2005. The A allele frequency in our control group was 0.14 compared to 0.21 calculated in the healthy white population evaluated in Aydin’s study [27]. The polymorphism was not included among non-coding loci significantly associated with QRS in the SNP meta-analysis [18]. Analysis of the polymorphism IVS9-3 C/A using the ESEfinder 3.0 program showed that the sequence change led to a change in the preferred acceptor splicing site. Near the newly formed acceptor splicing site, two strong exonic splicing enhancer (ESE) sites for non-splicing factors SRp40a and SC35 occur. As a result of the interaction of a cryptic acceptor site with a strong enhancer of splicing, a large insertion to final product of splicing is formed. This non-coding polymorphism is in tight linkage disequilibrium with H558R in the coding region of the SCN5A gene.

Recently, many investigators have suggested that rare variants in the coding regions of ion channel genes account for only a small fraction of SCDs [28]. A multi-centric study of 2111 patients with Brugada syndrome risk found 293 distinct variants of SCN5A [26].

Mutations in non-coding regions of SCN5A and KCNH2 have been linked to Brugada syndrome and long QT syndrome, respectively [2].

Recently, genome-wide association studies (GWASs) confirmed the strong association between single-nucleotide polymorphisms and some electric, structural and functional phenotypes of the cardiovascular system [29,30].

It is accepted that the range of variants in both the coding and non-coding regions of the genome, both rare alleles and polymorphisms, may influence cardiac electrophysiology [31]. Finally, the whole variability of the candidate genes must be included to obtain a real picture of the genetic background of complex diseases such as CAD.

It is questionable why the stenosis of a. coronaria dextran (not stenoses of other coronary arteries) is related to the SCN5A CCAA double genotype. In the literature, we found the first case of a congenital single coronary artery syndrome combined with DCM and with the SCN5A C.1858C > T (P.arg620Cys) mutation [32]. In one case report of the c. 664 C/T variant of SCN5A with a congenital absence of the a. coronaria dextra, patent foramen ovale together with ischemic stroke was reported [29]. Interestingly, SCN5A is 1 of 316 human fetal heart-specific genes which are known to be overexpressed in congenital heart diseases [33].

While many studies have provided deeper insight into the dysfunction and dysregulation of *SCN5A* and Nav1.5, it has become increasingly clear that sodium channel distribution, function and regulation is more complicated than assumed until now. Nav1.5-based channels display previously unrecognized non-electrogenic actions and may impact cardiac structural integrity, thereby also potentially affecting arrhythmogenesis. Moreover, *SCN5A* and Nav1.5 are expressed in many other cell types as well as in various extracardiac tissues, where their functional role is increasingly studied. *SCN5A* mutations have now been associated with (sudden unexpected death in) epilepsy and gastrointestinal disorders. The role of Nav1.5 in smooth muscle cell (SMC) function, cancer, innate immune response and inflammation has been reported [34].

Our study brings new results about the importance of germ cell polymorphisms in the SCN5A gene in patients with severe non-long QT arrhythmias and coronary artery disease. In all patients, some significant stenosis of the coronary artery was present which was associated with two SCN5A polymorphisms. On the contrary, the polymorphisms were not associated with arrhythmias in these patients. We present a highly sensitive and specific association of two polymorphisms in SCN5A with significant coronary artery stenoses in patients with potentially fatal ventricular arrhythmias. Thus, SCN5A gene polymorphic variants may form a part of the germ cell gene predisposition to ischemia. The loci, which were identified for CAD from a primary meta-analysis, do not include SCN5A gene variants [35]. The results seem to reflect a complicated pattern of germ cell rare allele/polymorphism combinations which participate in the development/severity of cardiac syndromes related to arrhythmia and ischemia [35].

A limitation of this study is the relatively low number of highly selected patients, which is to some extent equilibrated by high values of sensitivity/specificity of the calculated results. As usual, a higher number of similar patients should be evaluated for a final discussion about our results. Generally, large deeply phenotyped cohorts of patients are necessary to obtain clinically useful biomarkers for complex diseases.

## Figures and Tables

**Table 1:** Clinical statistics of parameters characterizing patients with severe arrhythmias.

	N = 32
**LV hypertrophy**	48%
Arrhythmia type A/A2/B/B2 *	53%/4%/34%/9%
**1 vessel disease**	31%
**2–3 vessel disease**	69%
**Ramus interventricularis anterior afflicted**	72%
**Ramus circumflexus afflicted**	59%
**Arteria coronaria dextra (ACD) afflicted**	69%

* A = ventricular fibrillation, EF ≤ 40%; A2 = ventricular flutter, EF ≤ 40%; B = ventricular fibrillation, EF > 40%; B2 = ventricular flutter, EF > 40%.

**Table 2:** Arrhythmia, 1 coronary vessel significant stenosis (1VS-1) and 2–3 coronary vessels significant stenosis (MVS-1).

Arrhythmia	1V-0	1VS-1	Comparison	MVS-0	MVS-1	Comparison
A	15	2	(A + A2) vs. (B + B2): *p* = 0.008 OR = 10.67 (95% CI 1.74–65.27) for (B + B2) and 1VD-1 Sensitivity 0.800 Specificity 0.727 Power test 0.708	2	15	(A + A2) vs. (B + B2): *p* = 0.008 OR = 10.67 (95% CI 1.74–65.27) for (A + A2) and MVS-1 Sensitivity 0.727 Specificity 0.800 Power test 0.708
A2	1	0		0	1	
B	4	7		7	4	
B2	2	1		1	2	
All Grps	22	10		10	22	

A = ventricular fibrillation, EF ≤ 40%; A2 = ventricular flutter, EF ≤ 40%; B = ventricular fibrillation, EF > 40%; B2 = ventricular flutter, EF > 40%.

**Table 3:** Case–control differences in two SCN5A polymorphisms.

*SCN5A* Exon/Intron	Nucleotide Change	Amino Acid Change	Region	Patients (N = 32) MAF	Number of Genotypes	Controls (N = 87) MAF	Number of Genotypes	Pg *	Pa **
Intron 9	IVS9-3c>a	-	-	20.3%	CC = 19 CA = 13 AA = 0	21.3%	CC = 51 CA = 35 AA = 1	0.831	0.873
Exon 12	A1673G	H558R	DI-DII	21.9%	AA = 18 AG = 14 GG = 0	27.0%	AA = 45 AG = 37 GG = 5	0.380	0.421

Pg * = probability of genotype distribution difference; Pa ** = probability of allelic frequencies difference.

**Table 4:** Two SCN5A polymorphisms and 2 or 3 coronary vessel significant stenosis (MVS-1) in patients with severe arrhythmia.

SCN5A Polymorphism	MVS-0	MVS-1	Comparison
**IVS9-3C/A**			
IVS9-3CC	3	16	*p* = 0.03 OR = 6.22 (95% CI 1.20–32.27) for CC genotype and MVS-1 Sensitivity 0.727 Specificity 0.700 Power test 0.478
IVS9-3C/A	7	6	
All Grps	10	22	
**A1673G**			
A1673A	3	15	*p* = 0.05 OR = 5 (95% CI 0.99–25.34) for AA genotype and MVS-1 Sensitivity 0.682 Specificity 0.700 Power test 0.359
A1673G	7	7	
All Grps	10	22	
**Double genotype of IVS9-3C/A with A1673G**			
CCAA	3	15	*p* = 0.05 OR = 5 (95% CI 0.99–25.34) for CCAA double genotype and MVS-1 Sensitivity 0.682 Specificity 0.700 Power test 0.359
CAAG	7	6	
CCAG	0	1	

**Table 5:** Presence of arteria coronaria dextra (ACD) affliction, multiple vascular stenosis (MVS) and SCN5A double genotypes.

ACD Afflicted	MVS	CCAA	CAAG	CCAG	Raw Σ
0	0	0	7	0	7
0	1	3	0	0	3
Σ1		3	7	0	10
1	0	3	0	0	3
	1	12	6	1	19
Σ2		15	6	1	22
All		18	13	1	32

0 = no affliction. 1 = affliction. Σ1 vs. Σ2: 3/7/0 vs. 15/6/1, *p* = 0.05, OR=5 (95% CI 0.99–25.34), sensitivity for CCAA = 0.70, specificity 0.682, power test 0.359. 00 vs. 01: 0/7 vs. 3/0, *p* = 0.008, sensitivity for CCAA = 1, specificity 1. 00 vs. 10: 0/7 vs. 3/0, *p* = 0.008, sensitivity for CCAA = 1, specificity 1. 00 vs. 11: 0/7 vs. 12/7, *p* = 0.005, sensitivity for CCAA = 0.631, specificity 1.

## Data Availability

Data are contained within the article.

## References

[1] Naik N., Yadav R. (2010). Genetics of sudden death. Indian J. Med. Res..

[2] Bezzina C.R., Lahrouchi N., Priori S.G. (2015). Genetics of sudden cardiac death. Circ. Res..

[3] Ruan Y., Liu N., Priori S.G. (2009). Sodium channel mutations and arrhythmias. Nat. Rev. Cardiol..

[4] Wilde A.A.M., Amin A.S. (2018). Clinical Spectrum of SCN5A Mutations: Long QT Syndrome, Brugada Syndrome, and Cardiomyopathy. JACC Clin. Electrophysiol..

[5] Tester D.J., Ackerman M.J. (2014). Genetics of long QT syndrome. Methodist Debakey Cardiovasc. J..

[6] Mazzaccara C., Limongelli G., Petretta M., Vastarella R., Pacileo G., Bonaduce D., Salvatore F., Frisso G. (2018). A common polymorphism in the SCN5A gene is associated with dilated cardiomyopathy. J. Cardiovasc. Med..

[7] Schwartz P.J., Crotti L., Insolia R. (2012). Long-QT syndrome: From genetics to management. Circ. Arrhythm. Electrophysiol..

[8] Ortiz-Bonnin B., Rinné S., Moss R., Streit A.K., Scharf M., Richter K., Stöber A., Pfeufer A., Seemann G., Kääb S. (2016). Electrophysiological characterization of a large set of novel variants in the SCN5A-gene: Identification of novel LQTS3 and BrS mutations. Pflugers Arch..

[9] Zaklyazminskaya E., Dzemeshkevich S. (2016). The role of mutations in the SCN5A gene in cardiomyopathies. Biochim. Biophys. Acta.

[10] Moss A.J., Schwartz P.J., Crampton R.S., Tzivoni D., Locati E.H., MacCluer J., Hall W.J., Weitkamp L., Vincent G.M., Garson A. (1991). The long QT syndrome. Prospective longitudinal study of 328 families. Circulation.

[11] Daimi H., Lozano-Velasco E., Aranega A., Franco D. (2022). Genomic and Non-Genomic Regulatory Mechanisms of the Cardiac Sodium Channel in Cardiac Arrhythmias. Int. J. Mol. Sci..

[12] Oliva A., Hu D., Viskin S., Carrier T., Cordeiro J.M., Barajas-Martinez H., Wu Y., Burashnikov E., Brugada R., Rosso R. (2009). SCN5A mutation associated with acute myocardial infarction. Leg. Med..

[13] Severino P., D’Amato A., Pucci M., Infusino F., Birtolo L.I., Mariani M.V., Lavalle C., Maestrini V., Mancone M., Fedele F. (2020). Ischemic Heart Disease and Heart Failure: Role of Coronary Ion Channels. Int. J. Mol. Sci..

[14] Severino P., D’Amato A., Pucci M., Infusino F., Adamo F., Birtolo L.I., Netti L., Montefusco G., Chimenti C., Lavalle C. (2020). Ischemic Heart Disease Pathophysiology Paradigms Overview: From Plaque Activation to Microvascular Dysfunction. Int. J. Mol. Sci..

[15] Raudenská M. Mutační Analýza Kandidátních Genů LQTS. Brno, 2010. Disertační Práce. Masarykova Univerzita, Přírodovědecká Fakulta. Vedoucí Práce RNDr. Ing. Karel Chroust, Ph.D. https://theses.cz/id/y577iy/.

[16] Liu X., Shi J., Xiao P. (2018). Associations between common ion channel single nucleotide polymorphisms and sudden cardiac death in adults: A MOOSE-compliant meta-analysis. Medicine.

[17] Marcsa B., Dénes R., Vörös K., Rácz G., Sasvári-Székely M., Rónai Z., Törő K., Keszler G. (2015). A Common Polymorphism of the Human Cardiac Sodium Channel α Subunit (SCN5A) Gene Is Associated with Sudden Cardiac Death in Chronic Ischemic Heart Disease. PLoS ONE.

[18] Sotoodehnia N., Isaacs A., de Bakker P.I., Dörr M., Newton-Cheh C., Nolte I.M., van der Harst P., Müller M., Eijgelsheim M., Alonso A. (2010). Common variants in 22 loci are associated with QRS duration and cardiac ventricular conduction. Nat. Genet..

[19] Ackerman M.J., Splawski I., Makielski J.C., Tester D.J., Will M.L., Timothy K.W., Keating M.T., Jones G., Chadha M., Burrow C.R. (2004). Spectrum and prevalence of cardiac sodium channel variants among black, white, Asian, and Hispanic individuals: Implications for arrhythmogenic susceptibility and Brugada/long QT syndrome genetic testing. Heart Rhythm..

[20] Liu J., Yao F., Han K., Chai J., Tian D., Zhang J., Wang R., Li W., Shen Y., Ma Y. (2021). Relationship between SCN5A gene H558R polymorphism and atrial fibrillation in Tibetan and Han nationalities at high altitude. Medicine.

[21] Magnani J.W., Brody J.A., Prins B.P., Arking D.E., Lin H., Yin X., Liu C.T., Morrison A.C., Zhang F., Spector T.D. (2014). Sequencing of SCN5A identifies rare and common variants associated with cardiac conduction: Cohorts for Heart and Aging Research in Genomic Epidemiology (CHARGE) Consortium. Circ. Cardiovasc. Genet..

[22] Chen J.Z., Xie X.D., Wang X.X., Tao M., Shang Y.P., Guo X.G. (2004). Single nucleotide polymorphisms of the SCN5A gene in Han Chinese and their relation with Brugada syndrome. Chin. Med. J..

[23] Viswanathan P.C., Benson D.W., Balser J.R. (2003). A common SCN5A polymorphism modulates the biophysical effects of an SCN5A mutation. J. Clin. Investig..

[24] Matsumura H., Nakano Y., Ochi H., Onohara Y., Sairaku A., Tokuyama T., Tomomori S., Motoda C., Amioka M., Hironobe N. (2017). H558R, a common SCN5A polymorphism, modifies the clinical phenotype of Brugada syndrome by modulating DNA methylation of SCN5A promoters. J. Biomed. Sci..

[25] Ye B., Valdivia C.R., Ackerman M.J., Makielski J.C. (2003). A common human SCN5A polymorphism modifies expression of an arrhythmia causing mutation. Physiol. Genom..

[26] Verkerk A.O., Amin A.S., Remme C.A. (2018). Disease Modifiers of Inherited *SCN5A* Channelopathy. Front. Cardiovasc. Med..

[27] Aydin A., Bähring S., Dahm S., Guenther U.P., Uhlmann R., Busjahn A., Luft F.C. (2005). Single nucleotide polymorphism map of five long-QT genes. J. Mol. Med..

[28] Glazer A.M., Wada Y., Li B., Muhammad A., Kalash O.R., O’Neill M.J., Shields T., Hall L., Short L., Blair M.A. (2020). High-Throughput Reclassification of SCN5A Variants. Am. J. Hum. Genet..

[29] Pirruccello J.P., Di Achille P., Nauffal V., Nekoui M., Friedman S.F., Klarqvist M.D.R., Chaffin M.D., Weng L.-C., Cunningham J.W., Khurshid S. (2022). Genetic analysis of right heart structure and function in 40,000 people. Nat. Genet..

[30] Hu X., Kong J., Niu T., Chen L., Yang J. (2023). Single coronary artery presenting dilated cardiomyopathy and hyperlipidemia with the *SCN5A* and *APOA5* gene mutation: A case report and review of the literature. Front. Cardiovasc. Med..

[31] Albert C.M., Nam E.G., Rimm E.B., Jin H.W., Hajjar R.J., Hunter D.J., MacRae C.A., Ellinor P.T. (2008). Cardiac sodium channel gene variants and sudden cardiac death in women. Circulation.

[32] Katsaras D., Sanjeev Kumar B.T., Patel B., Chalil S., Abozguia K. (2021). A 59-Year-Old Woman with Familial Brugada Syndrome and the c.664C>T Variant of the Sodium Voltage-Gated Channel α Subunit 5 (SCN5A) Gene, Accompanied by Congenital Absence of the Right Coronary Artery, Patent Foramen Ovale, and Ischemic Stroke. Am. J. Case Rep..

[33] Wang B., You G., Fu Q. (2017). Human fetal heart specific coexpression network involves congenital heart disease/defect candidate genes. Sci. Rep..

[34] Remme C.A. (2023). *SCN5A* channelopathy: Arrhythmia, cardiomyopathy, epilepsy and beyond. Philos. Trans. R Soc. Lond B Biol. Sci..

[35] Aragam K.G., Jiang T., Goel A., Kanoni S., Wolford B.N., Atri D.S., Weeks E.M., Wang M., Hindy G., Zhou W. (2022). Discovery and systematic characterization of risk variants and genes for coronary artery disease in over a million participants. Nat. Genet..

